# BICAR: A New Algorithm for Multiresolution Spatiotemporal Data Fusion

**DOI:** 10.1371/journal.pone.0050268

**Published:** 2012-11-28

**Authors:** Kevin S. Brown, Scott T. Grafton, Jean M. Carlson

**Affiliations:** 1 Department of Physics, University of California, Santa Barbara, California, United States of America; 2 Institute for Collaborative Biotechnologies, University of California, Santa Barbara, California, United States of America; 3 Chemical, Materials, and Biomolecular Engineering, University of Connecticut, Storrs, Connecticut, United States of America; 4 Department of Marine Sciences, University of Connecticut, Groton, Connecticut, United States of America; 5 Department of Psychological and Brain Sciences, University of California, Santa Barbara, California, United States of America; University of Ulm, Germany

## Abstract

We introduce a method for spatiotemporal data fusion and demonstrate its performance on three constructed data sets: one entirely simulated, one with temporal speech signals and simulated spatial images, and another with recorded music time series and astronomical images defining the spatial patterns. Each case study is constructed to present specific challenges to test the method and demonstrate its capabilities. Our algorithm, BICAR (**B**idirectional **I**ndependent **C**omponent **A**veraged **R**epresentation), is based on independent component analysis (ICA) and extracts pairs of temporal and spatial sources from two data matrices with arbitrarily different spatiotemporal resolution. We pair the temporal and spatial sources using a physical transfer function that connects the dynamics of the two. BICAR produces a hierarchy of sources ranked according to reproducibility; we show that sources which are more reproducible are more similar to true (known) sources. BICAR is robust to added noise, even in a “worst case” scenario where all physical sources are equally noisy. BICAR is also relatively robust to misspecification of the transfer function. BICAR holds promise as a useful data-driven assimilation method in neuroscience, earth science, astronomy, and other signal processing domains.

## Introduction

Combining multiple data sets with complementary spatial and temporal resolution in order to obtain an integrated view of a process of interest with high spatiotemporal resolution is a difficult problem that arises in many disparate contexts. Two examples are (i) combining satellite measurements (slow, dense) with ground-based sensors (fast, sparse) in earth science [1]–[4], and (ii) simultaneous electroencephalographic (fast, sparse) and functional magnetic resonance (slow, dense) measurements of human brain activity [5]–[7]. When the data are generated by a common process for which a good model exists, for example in oceanic state estimation [8] and atmospheric science [9], it is possible to “assimilate” the model and the data using least squares techniques [10]–[12]. However, doing this for multiple sets of measurements can be difficult, and when no reliable equations of motion exist, no such data assimilation is possible. For these reasons, it is desirable to develop purely data-driven techniques that aim to co-associate features in two sets of dynamical measurements with vastly different resolution.

Independent Component Analysis (ICA) is a nonlinear technique used to “unmix” spatial and temporal data into statistically independent sources and corresponding mixing (or unmixing) coefficients that relate the degree to which each (statistical) source is present in each (real) sensor [13]–[15]. While originally developed to solve the so-called cocktail party problem [13], in which the goal is to separate the voices of individual speakers from mixed room recordings, ICA has become an extremely powerful and popular model reduction technique, with applications in neuroscience [16], [17], earth science [18], and astronomy [19]. One of the most popular algorithms for performing this unmixing is FastICA [20]. However, most ICA algorithms and analyses (including FastICA) suffer from two difficulties: (i) unmixing to statistically independent sources is a difficult nonlinear optimization problem which can show sensitivity to the starting guess for the mixing matrix and can become trapped in local optima, and (ii) in noisy data where the sources of interest may represent only a small fraction of the total variance in the data set (for example electroencephalographic data) it can be difficult to objectively rank the ICA sources. Both of these difficulties contrast with the far simpler case of unmixing to linearly decorrelated, rather than statistically independent, sources. Doing so is a linear algebra problem with a single global optimum, and goes under the name principal component analysis (PCA) [21], empirical orthogonal function (EOF) analysis [22], the discrete Karhunen-Loeve transform (KLT) [23], or proper orthogonal decomposition (POD) [24], depending upon the field the user of the technique hails from (generally, statistics, geophysics, mathematics, and engineering, respectively).

In order to attempt to surmount the difficulties in decomposition and component interpretation described above, several investigators have advanced proposals to make ICA more robust. These suggestions include clustering components obtained from multiple ICA runs [25] and analyzing ICA sources for peaks at known frequencies [26]. Such peaks could occur if the system is being forced by some other known or measured process. Others have advanced the idea that the concept of reproducibility, the degree to which a similar-looking source occurs repeatedly in multiple ICA runs, could simultaneously address the shortfalls in (i) and (ii) above [27]. Components that are produced in multiple ICA runs from different starting conditions represent particularly strong signals in the data, and ranking those components by reproducibility aids in interpretability. This is the idea behind the RAICAR algorithm [27], which produces reproducible components for a single data matrix of interest. Even if it is possible to rank the components by other means, reproducibility can always form an additional comparative axis that indicates the order in which one should look at ICA sources and quantifies the amount of trust that should be placed in those sources.

We extend the concepts in Ref. [27] to develop BICAR, a new algorithm to extract *paired* sources of interest from two sets of sensor data with vastly different (hopefully complementary) degrees of sensor coverage and sampling rate. This problem has attracted a good deal of attention in human neuroimaging, in which a variety of attempts, some of which use ICA [28], [29], have been made to distill shared variability from multiple measurements. If one wants to decompose these data “all at once” [6], [30]–[32] a series of essentially arbitrary resamplings must be made in order to bring at least one of the matrix dimensions of each data set to conformability. An additional contribution to the ICA-based multimodal fusion literature comes from Multimodal Independent Component Analysis (MICA) [33], which attempts to solve an augmented stochastic optimization problem incorporating independence of sources within data sets and statistical dependence across data sets. However, MICA also requires conformable matrix dimensions be obtained via resampling, as direct source-source correlations are required for data preconditioning and the subsequent minimization problem. In addition, MICA by itself does nothing to alleviate the component interpretation problem described above. Our method does not require accidental conformability of the space and time dimensions in either data set, and BICAR simultaneously addresses the problem of component reliability. We also preserve the physical link between the two data sets by assuming that the spatial data represents a transformed (filtered), downsampled version of the temporal data.

The purpose of this paper is threefold: (a) to introduce the BICAR algorithm, (b) to show it works in a quasi-simulation context with data of real-world complexity, and (c) to explore robustness of the algorithm to assumptions. In what follows we describe BICAR in detail, and consider its performance on three constructed data sets of varying difficulty: one set of simulated data, one which employs temporal speech signals and simulated spatial images, and a third which uses musical time series paired with astronomical images. We show that BICAR recovers the true sources that constitute the data even when both data matrices have been corrupted by Gaussian noise and the transfer function that connects the two data matrices is imperfectly known.

## Methods

### Algorithm

**Figure 1:**
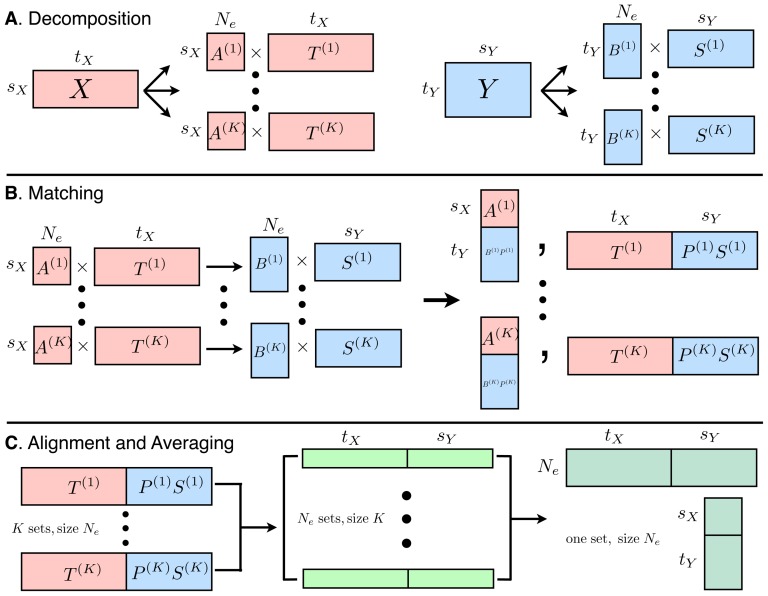
Schematic for the BICAR algorithm. This diagram illustrates the change in size and number of matrices during the steps of BICAR. **A**. During the decomposition step, both the temporal data 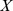 and spatial data 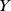 are decomposed into 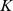 sets of 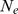 sources, where 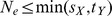. The other necessary assumptions, 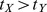 and 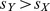, are also schematically depicted. **B**. The matching step can be viewed as a concatenation, in which 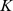 sets of 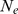 super-sources and corresponding mixing elements are obtained. This step calculates permutations of the spatial decompositions to give the groupings. **C**. In the alignment step, the 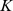 sets of 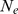 matched sources are sorted into 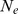 sets of 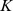 super-sources and their corresponding mixing matrices. Here 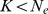, but there need be no particular relationship between these two quantities. Finally, after averaging and reproducibility calculations, matrices whose sizes correspond exactly with one of the 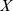 decompositions and one of the 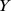 decompositions shown in **A** remain.

The BICAR pipeline is summarized in Fig. 1. BICAR proceeds under relatively mild assumptions. The first is that there are two data matrices, 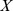 and 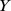. 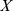 is of size 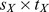, and 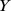 of size 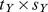. The 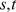 notation has been deliberately chosen to indicate space and time. The sizes are assumed to follow the relationship 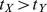 and 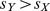, that is, the 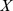 matrix (the “temporal” data) has high temporal resolution and coarse spatial resolution, while the 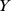 matrix (the “spatial” data) has high spatial resolution and coarse temporal resolution. (In practice, usually 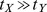 and 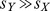.) None of these matrix dimensions need be equal. In what follows, we describe each of BICAR's steps in detail and give an equivalent pseudocode representation at the end of each subsection. The pseudocode is to be understood as pedagogical, in which efficiency has been sacrificed for explanatory clarity.

### Unmixing

BICAR begins by performing stochastic ICA 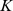 times on each data matrix separately (see Figure 1A). 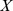 and 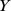 are arranged in such a way that ICA produces independent *temporal* sources for 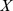 (and a mixing matrix), and independent *spatial* sources for 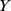 (again, with a mixing matrix). Specifically, each ICA decomposition uses the linear source separation model
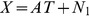
(1)

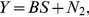
(2)where 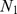 and 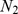 are noise, 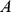 and 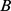 are mixing matrices, and 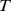 and 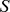 are matrices of temporal and spatial sources, respectively, and the 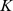 sets of decompositions are denoted as follows:
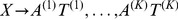
(3)

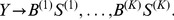
(4)



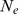
 sources are extracted from each of 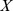 and 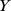; 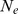 may be as large as 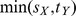. Usually, 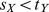, which, if 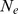 were as large as possible, would generate a full-rank decomposition of 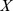 and a reduced-rank one of 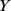. One could alternately ask for fewer sources, using a variety of criteria to reduce the number of sources to less than 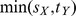.[34]–[36].


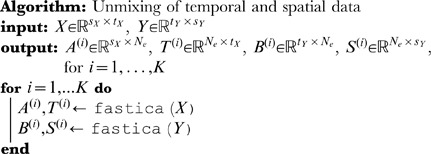


### Matching

After decomposition, a match step is used that associates features in the temporal decompositions with features in the spatial decompositions (see Figure 1B). Specifically, the temporal mixing coefficients (columns) of the 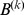 matrices are assumed to be functions of the temporal sources (rows) of the 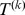 matrices. For the numerical experiments in this paper, a particular form is assumed, which is convolution followed by decimation. Specifically,
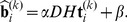
(5)

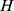
 is a circulant matrix representation of the convolving function (for example a lowpass filter with delay), 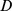 is a decimation operator of size 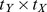, and 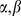 are scalars representing potentially unknown unit transformations. Both 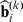 and 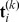 are to be understood as column vectors. With reference to 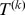 and 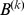 in Eqns. 3 and 4, 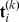 is the transpose of one of the *rows* of 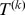 and 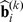 will be compared to the *columns* of 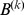.

The matching step proceeds as follows. For 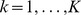, each row of 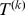 is transformed according to Eqn. 5 and correlated with all columns of 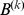. The columns of 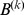 are then paired with the rows of 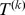 without degeneracy: the pair with the largest absolute correlation are paired and removed from the pool, and the process is repeated until all temporal sources in realization 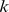 have a pair in the spatial realization. This pairing with 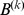 automatically pairs 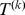 with the spatial sources 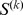, as column 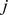 in 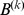 corresponds with source (row) 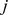 in 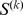. This process can be envisioned as creating a row permutation matrix 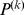, that when (left) multiplying 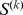 the spatial sources are ordered so they are paired, row-by-row, with their best matching row in 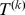, according to Eqn. 5.


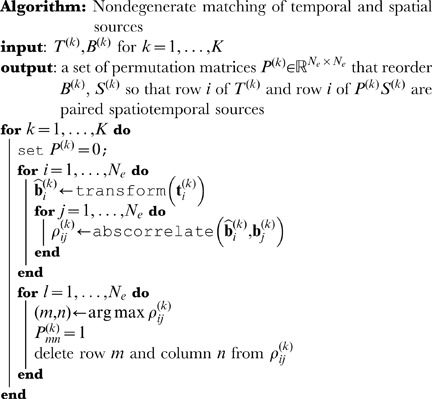


### Cross-realization correlations

Once temporal and spatial sources are matched, a set of 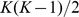 cross-realization correlation matrices (CRCMs) is computed [27]. These matrices represent the absolute value of the correlation coefficients between all *paired* sources in two realizations. In BICAR, each realization contains *two* sets of sources that have been linked via the matching step. The CRCMs are therefore computed as follows:
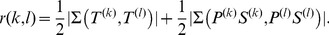
(6)In this equation, each 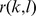 is a 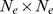 matrix of absolute cross correlations. We have used the symbol 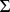 to represent a single cross-correlation matrix; for example, the 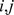 element of 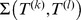 consists of the Pearson correlation between row 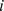 in 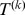 and row 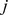 in 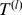. Absolute value bars are understood to be applied element-wise to each cross correlation. BICAR source similarity is computed in both time and space, hence the presence of two terms, one measuring correlations among 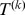 and 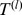 (time) and one for 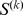 and 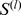 (space). Multiplication by 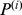 in the second term reorders the rows of 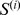 so that it is in the matching row order described above. Alternatively, one can think of computing 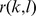 as correlating sets of super-sources, in which 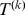 is concatenated with 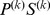. The calculation of the set of 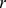 matrices in Eqn. 6 assumes that 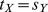. If this is not the case, the factors of 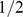 can be replaced with weights 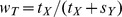 and 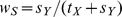.


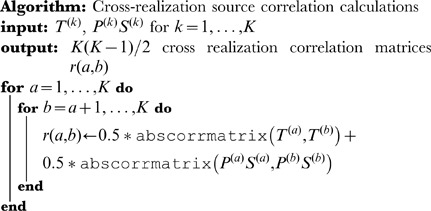


### Source alignment

The goal of searching the CRCMs and aligning similar components is to resort the 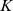 sets of 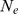 super-sources into 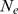 sets of 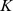 super-sources. Once this is done, these sets will be averaged to obtain an ICA-like decomposition that uses all 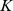 realizations (see Figure 1C). This sorting step proceeds as follows. Once the set of matrices in Eqn. 6 are calculated, they are searched exactly as in the RAICAR algorithm [27]; pseudocode is therefore suppressed for this step. Briefly, the largest element among all the matrices is selected first. Denote the location of this element as 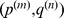, that is, row 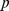 and column 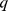 in the matrix coming from cross-correlations between realizations 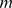 and 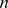. This element represents the two most similar sources, out of all cross realization pairs. After finding this element, an additional 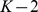 sources are selected to pair with source 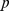 from realization 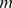 and 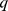 from realization 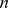. This is done by searching row 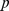 of matrices 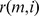 for 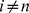 and column 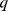 of matrices 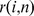 for 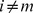 for their respective maxima. In cases where the row and column maxima for realization 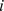 are identical, the super-source corresponding to that location is added to the growing super-component. If they are not equal, the source from the realization with the larger of the two correlation values is added to the growing super-component. Once all realizations have been searched via the CRCMs, one super-source from each component has been extracted. The rows and columns in each 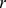 matrix associated with these extracted super-sources are then deleted, and the process is repeated 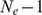 more times.

### Sign canonicalization

Alignment has resorted the super-sources so that, rather than 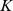 sets of 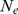 sources, there are now 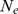 sets of 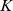 sources, arranged to be maximally within-group similar (again see Figure 1C). Before averaging over the 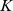 realizations in each group, it is necessary to deal with a sign problem. While ICA is guaranteed to produce a set of sources and mixing matrix that reconstruct the data matrix, one can easily flip the sign of one or more sources and the signs of the corresponding columns in the mixing matrix and leave the reconstructed data matrix invariant. Because sources are aligned using *absolute* Pearson correlation coefficients, sources in one realization and their sign-reversed versions will be aggregated together. Therefore, before averaging and reproducibility calculation, a simple procedure is employed to attempt to “canonicalize” the signs. Recall that the first two sources added to one of the 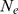 groups represent the two most similar sources remaining, across all realization-realization pairs. Therefore the sign of the first source is adopted as canonical, and the signed correlation of that source with the other 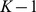 sources in its group are computed. For any correlations which are negative, flip the sign of both the source *and* the corresponding mixing matrix column, which has been carried along in the alignment process for both the temporal and spatial decompositions. This manipulation is repeated for all 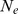 groups.


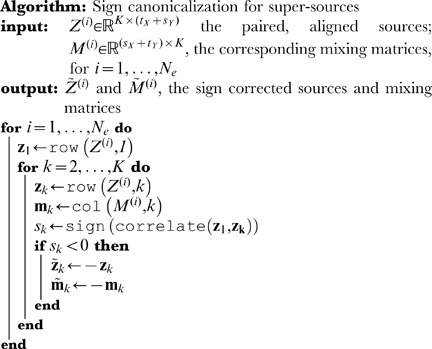


### BICAR source and reproducibility calculation

After canonicalization, the sources are combined as follows (see Figure 1C). This step has the effect of producing pseudo-realizations. The results have the same shape as the single ICA decompositions in Eqn. 2; however, the BICAR averaged sources are not in general true independent components, and they have a ranking in terms of reproducibility. We define the reproducibility of one of the 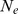 super-sources as the sum of the unique intra-group absolute cross-correlations divided by 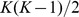. This places the reproducibility index 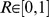. Each of the 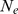 groups of 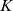 sources are then collapsed to one source by weighted averaging; the weight for each source is its average absolute cross-correlation with the other 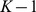 sources. The sources are then ranked in order of reproducibility.

### Numerical experiments

The primary considerations in constructing validation data are (a) to demonstrate BICAR's performance on data with “real-world” complexity and (b) to deliberately construct paired temporal and spatial data in a manner that avoids domain-specific attributes that would arise in neuroscience, earth science, or any other specific signal processing domain. Testing BICAR on data constructed and conjoined in a pre-specified way validates the capabilities and robustness of the algorithm in a situation where the underlying signals and their relationships are known exactly. A detailed description of the validation data and some comments on algorithm parameters follow.

### Hyperparameters

#### Number of realizations

The number 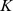 of ICA realizations in the unmixing step needs to be specified. 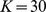 is used for all studies in this manuscript. The choice of 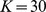 is motivated by the RAICAR


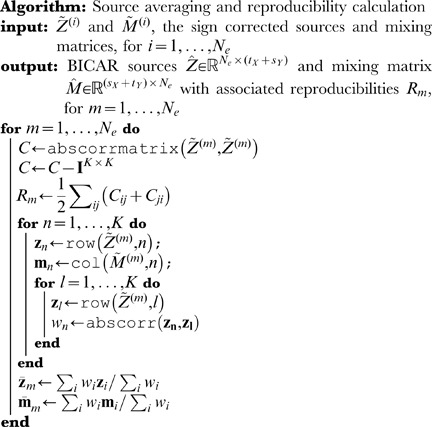


algorithm [27] (see particularly Figure 11 in that reference). While simulations to fine tune 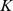 were not conducted for this study, doubling 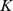 (to 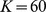) yielded no improvement in algorithm performance but imposed a substantial computational cost (not shown). Drastically reducing 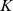 (to 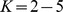) resulted in much poorer algorithm performance (not shown). This is to be expected since repeated estimations are a critical feature of BICAR.

#### Linkage between the spatial and temporal data

In our numerical experiments, we assume the temporal and spatial datasets are linked via convolution followed by decimation (see Eqn. 5). Many methods for downsampling real data are possible; for the numerical experiments in this paper, 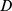 (the decimation matrix) is simply integer downsampling. While other methods could be used, they are not considered here. By using correlation to match the spatial and temporal sources, we can ignore the unknowns 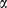 and 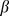. For most of the numerical experiments in this manuscript 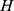 (the convolving function) is assumed to be known (see Mixing), but mismatches in 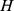 are also considered (see Results). BICAR is not confined to linking the datasets via only this transformation; the transformation between spatial and temporal data could be linear or nonlinear, and parametric or nonparametric (i. e. empirical filter coefficients). This transformation will likely depend on the data domain; see Mixing for details on 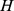 and its rationale. The important assumption is the connection between the 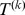 and 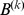 matrices, not the particular form of that connection.

#### Matching method

It is not essential to use the “online” matching method described here, in which paired decompositions are compared in turn; all-against-all matching is more computationally intensive but similar in procedure. Degenerate matching, in which different temporal sources are paired with the same spatial source, is also possible. In this study we consider only nondegenerate matching, in which each temporal source in each realization has a unique pair among the spatial sources.

### Temporal sources

#### BLOBS

**Figure 2:**
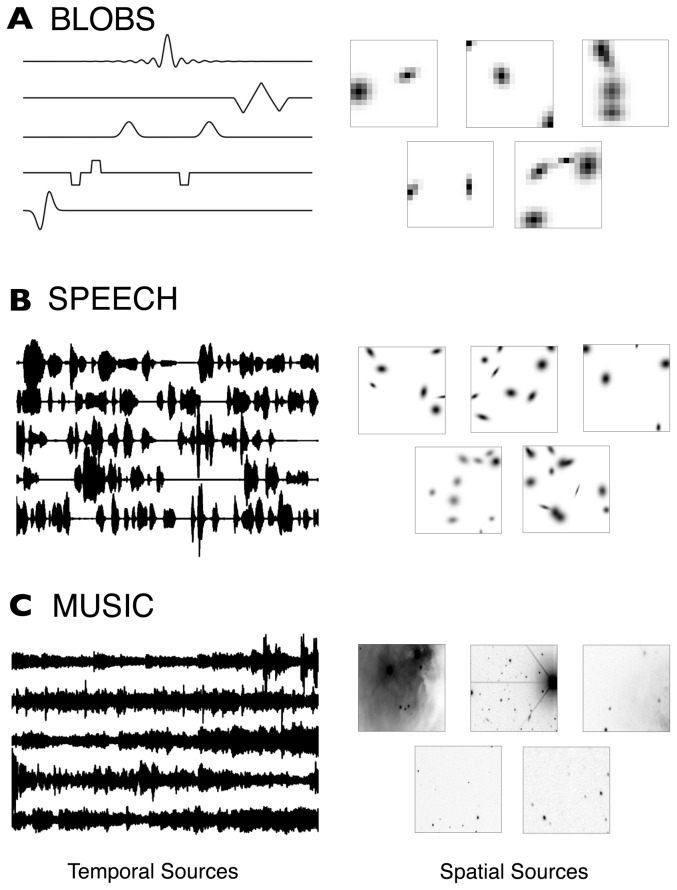
The three sets of temporal and spatial signals used in this study. For each of the three cases (**A**, **B**, and **C**) five representative temporal sources are shown at left and five representative spatial sources are shown at right; for details on source construction see “Methods.” The constructed spatial sources are shown in an astronomical convention, whereby darker color indicates higher image intensity (reversed grayscale). **A**. The BLOBS data set has temporal sources constructed from simple windows and spatial sources made from Gaussian blobs. **B**. The SPEECH data set has temporal sources extracted from five different public domain audiobooks, and spatial sources constructed of Gaussians. **C**. The MUSIC data set features temporal sources extracted from five different public domain live concerts, and spatial sources that are small frames extracted from much larger astronomical images from the Sloan Digital Sky Survey (http://www.sdss.org).

Five simple signals with limited temporal support and overlap were created and are shown in Figure 2A. The signals are composed of simple waveforms (sinusoidal, Blackman windows, Gaussian pulses, etc.). Each signal was designed to mimic a 1 second recording at 256 Hz. The signals were standardized to have zero mean and unit variance.

#### SPEECH

Five mp3 files were obtained from a repository of public domain audiobooks (librivox.org) and downsampled to 2.75 kHz. The works used were “Flatland,” by Edwin A. Abbott, The “Confessions” of St. Augustine, “Huckleberry Finn” by Mark Twain, Herman Mellvile's “Moby Dick”, and “History of the Peloponnesian War”, Book 5, by Thucydides. Each realization of this data pulled 128^2^ contiguous samples from a random location in the overall file, corresponding to a roughly six second block of speech. Each block was standardized to have zero mean and unit variance; a representative set of these speech signals is shown in Figure 2B.

#### MUSIC

Five mp3 files were obtained from a repository of public domain sound recordings (www.archive.org/details/etree). The artists used were Andrew Bird, Bela Fleck and the Flecktones, Cowboy Junkies, The Mekons, and The National. These two-channel recordings were averaged to monaural and downsampled to 2.75 kHz. From each processed recording, 128^2^ contiguous samples were extracted from a random location in the overall recording, corresponding to a roughly six second block out of several minutes of total recording time. Each block was then standardized to have zero mean and unit variance; a representative set is shown in Figure 2C.

### Spatial sources

#### BLOBS

Each simulated spatial source is a 16×16 pixel array, viewed as a pixelisation of 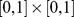. This image size was chosen to correspond to the number of samples in each simulated temporal source, described above. Each spatial source contains a random number of non-normalized Gaussians (between two and four) of the form
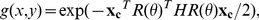
(7)where 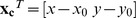, with a random center 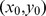 in 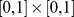, 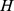 is a 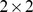 diagonal Hessian matrix with random entries in the range 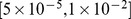, and 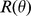 is the following 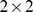 rotation matrix:
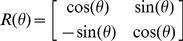
(8)As with the simulated temporal sources, each simulated spatial source was standardized. The data are shown in Figure 2A.

#### SPEECH

These spatial sources were constructed in an identical fashion to those in BLOBS, with the following differences: there are between four and fifteen Gaussians, the diagonal Hessian matrix has random elements in 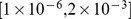, and the images are 128×128 pixels. A representative set of sources is shown in Figure 2B.

#### MUSIC

Five astronomical images were downloaded from the image gallery of the Sloan Digital Sky Survey (www.sdss.org). The images were of varying sizes, so they were all interpolated and downsampled to 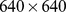 pixels. Each spatial source is a random 128×128 pixel subimage extracted from one of these images, one source per image. Thus the chosen sources are 4% of the total number of pixels in the downsampled image. Following extraction, the spatial sources were standardized as before; a representative set of images is shown in Figure 2C.

### Mixing

**Figure 3:**
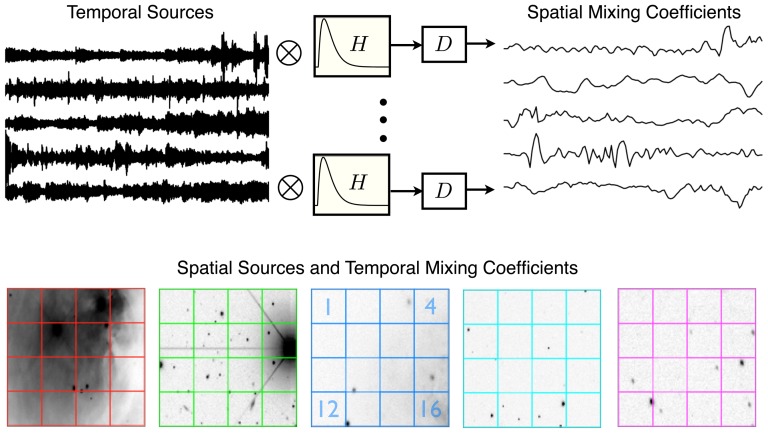
Mixing coefficients, shown for the MUSIC data set. At top, the process of transformation and decimation/downsampling of the temporal sources which leads to the spatial mixing coefficients (see Eqn. 5) is shown. At bottom, the temporal mixing coefficients are obtained by spatial downsampling; each image is divided into sixteen blocks, and the mean intensity in each block gives one column of the temporal mixing matrix. This process is repeated for each image, resulting in 16 mixtures of the five temporal sources. Four of these regions are numbered in blue in the third image, with the numbering suppressed in other images for clarity. Mixing coefficients for the BLOBS and SPEECH data sets are constructed analogously; see Methods for details.

A schematic showing the mixing of the spatial and temporal sources is shown in Fig. 3. 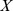 and 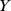 are assumed to be related via the transformation in Eqn. 5. A delayed, low-pass filtered version of the temporal sources was simulated by convolving with the following function
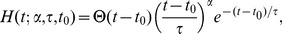
(9)where 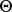 is the Heaviside function. This function has a single peak at 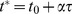. If not otherwise noted in the text, 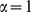, 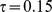, and 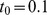. After this transformation, the resulting signals were further decimated by a factor of either 16 (BLOBS) or 128 (SPEECH, MUSIC). This delayed, filtered, decimated signal forms one set of mixing coefficients; there are five in total, one for each source.

To mix the temporal sources, the area occupied by the spatial sources was divided into sixteen blocks. One column of temporal mixing coefficients was obtained by computing average intensity values in those sixteen blocks. This was repeated for each spatial source, yielding a mixing matrix of size 16×5. This process is identical in BLOBS, SPEECH, and NOISE, although the SPEECH and NOISE blocks are larger because those images are larger (128 pixels on a side versus 16).

For the validation simulations, this transfer function was chosen a priori. It has a form (a low pass, delayed LTI filter) commonly observed in physical systems, including fMRI [37]–[39]. This particular transfer function was chosen for its familiarity, its generality, and because it is relatively easy to manipulate the function's shape parameters and thereby investigate robustness to TF misspecification. The TF will depend on the process being studied and could take a different form, including that of a nonlinear model linking the spatial and temporal data, in which case Eqn. 5 would need to be modified. Linking the simulations as described above allows validation of BICAR in a situation in which both the input data and the form of the linkage between the spatial and temporal data is known.

### Noise

After mixing of temporal and spatial sources, noise was added in a symmetric way to both data matrices, allowing a signal-to-noise (SNR) measure to be defined for each simulation. Both the noiseless temporal and spatial mixtures were first normalized such that the variance of each matrix was equal to unity. Then matrices of Gaussian random noise of the appropriate size with zero mean and variance 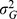 ranging from 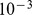 to 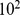 were added to the data matrices. The SNR of the resulting noisy data was defined as 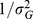.

### Reconstruction quality

A quality factor 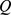 was defined for BICAR reconstructions as follows. Every BICAR source, consisting of paired temporal and spatial components, was absolute correlated with the five known noiseless sources. The resulting correlation matrix was searched for successive maxima and reduced in dimension by one unit at each step. Thus each BICAR source becomes associated with a unique best match among the known paired sources. Denoting the value for BICAR source 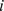 during this search by 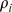, define 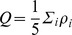, the average of the absolute correlations.


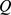
 alone does not uniquely inform us about the BICAR decomposition; one could obtain 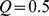 by having perfect matches from the temporal parts of the BICAR sources and terrible ones from the spatial portions or vice versa. In some cases it is useful to distinguish the contribution of 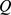 from temporal source similarity and the contribution coming from spatial source similarity. Hence sub-measures 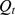 and 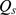 were also defined. These simply sum the pieces of the 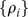 coming from absolute correlations between the temporal parts of joined sources (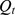) and similarly for the spatial pieces (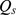). 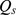 and 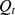 are both bounded above by 0.5, while 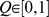.

## Results

**Figure 4:**
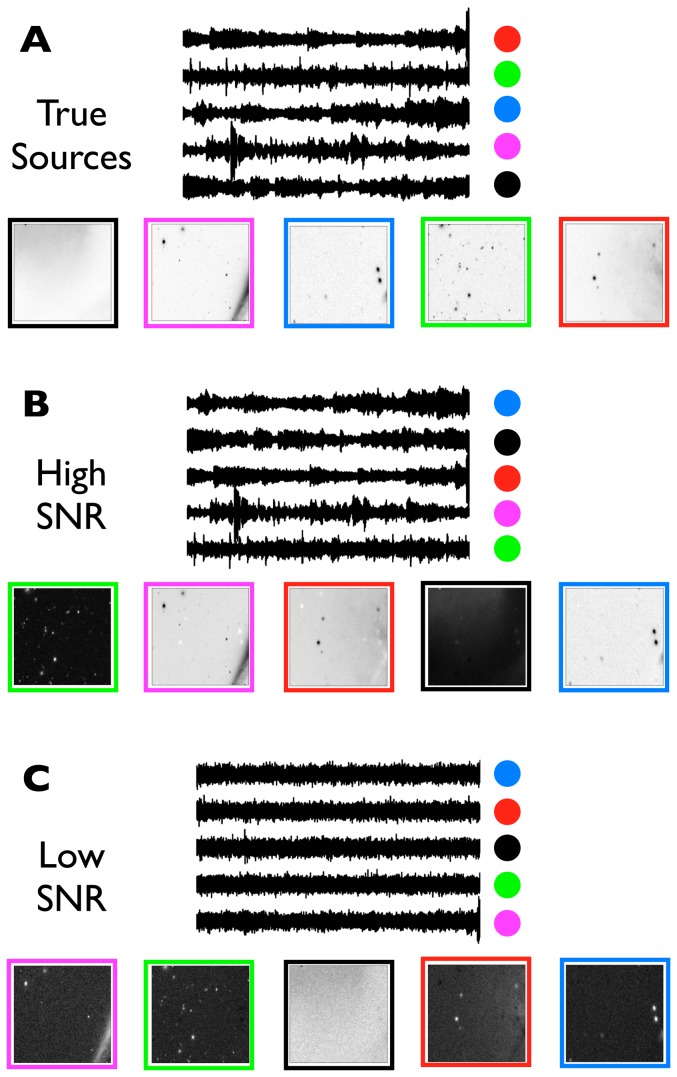
Low and high noise BICAR reconstructions for MUSIC data. **A**. The noiseless true sources. Colored dots and boxes have been used to show both the pairing between temporal and spatial sources and the association between BICAR sources in **B**, **C** and the true sources. For example, the temporal source on the bottom of the signal plot is paired with the leftmost spatial source in the image series. **B**. A BICAR reconstruction at low noise (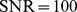). The colors have been assigned according to the best match with a true source; note that some sources have reversed signs (green and black), but pairing the BICAR sources with the true sources is quite easy. **C**. A high noise BICAR reconstruction (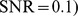. While features of the true spatial sources are evident in the BICAR sources, the temporal sources are pure noise.

Figure 4 illustrates sample BICAR reconstructions for the MUSIC data at both high and low signal-to-noise. Fig. 4A shows the true temporal and spatial sources; colored circles next to the temporal signals and colored boxes around the spatial signals give the source pairing. For example, the fourth temporal source from the bottom (green dot) has been paired with the fourth spatial source from the left (green box). Fig. 4B shows a BICAR reconstruction at low noise (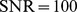). The colors now indicate both the temporal and spatial pairing, as well as the best true match for each BICAR source. Notice first that two of the spatial signals (first and fourth from the left) have had their signs reversed; this is no cause for concern, as the sign canonicalization procedure ensures consistent signs, but not necessarily the same sign as a true source. At this low noise level, it is easy to determine which BICAR source matches which true source; the BICAR sources strongly resemble the true sources.

Figure 4C shows a BICAR reconstruction at high noise (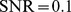). Once again, several spatial sources have reversed signs with respect to their true counterparts. Also note that the BICAR sources are much noisier than in the low noise case; the temporal sources are basically unrecognizable, but some of the features of the true spatial sources can still be seen in their BICAR equivalents - however they have begun to be spread across multiple sources. For the scenario we have constructed — all sources with identical signal-to-noise — it is entirely expected that the sources may become much noisier as the added noise increases. Since ICA reconstructs each data matrix (temporal and spatial) with little error, the added noise must go somewhere, either into the sources themselves or the mixing coefficients. Clearly, BICAR can help to reduce this noise via source averaging, at least in the case of the spatial sources in this example. There are additional remarks on this asymmetry between the temporal and spatial source quality below.

**Figure 5:**
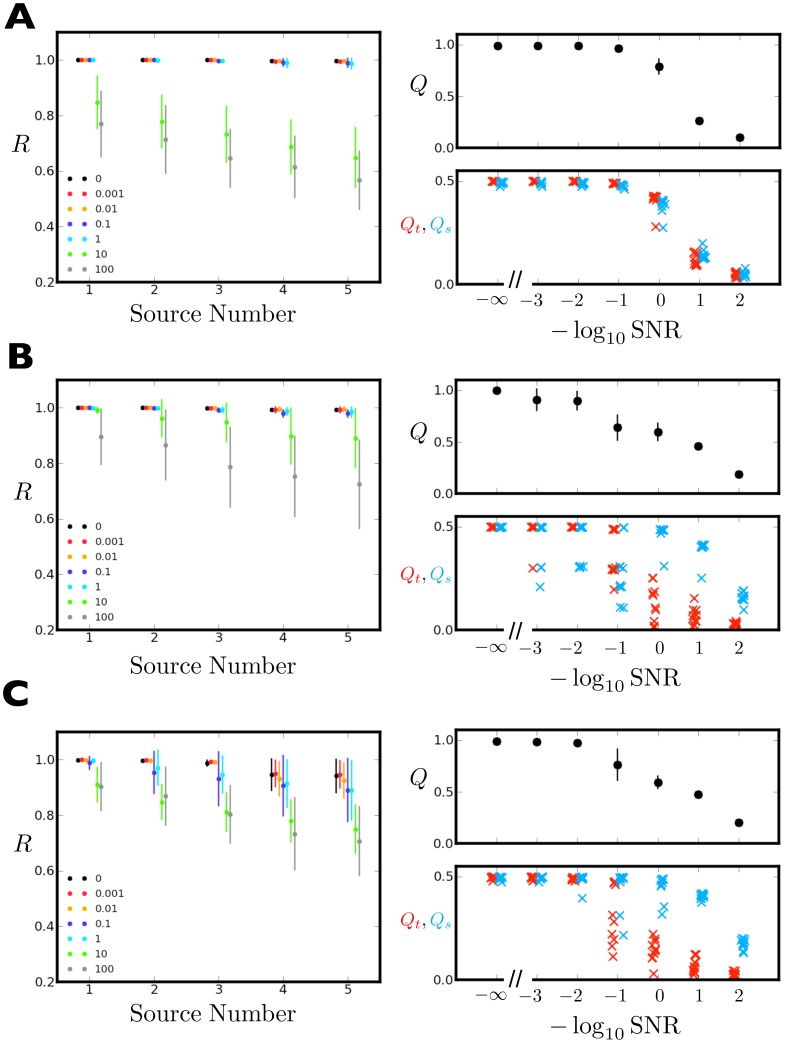
Mean reproducibility spectrum and quality index. **A**. BLOBS. **B**. SPEECH. **C**. MUSIC. Results are averaged over ten BICAR runs at each of seven different noise-to-signal ratios (1/SNR). In the reproducibility plots (left panels of **A**, **B**, and **C**), the values for each inverse SNR have been offset for clarity. All BICAR sources have been ranked in order of decreasing reproducibility before averages are computed. In the bottom right panels, the spatial (blue **x**) and temporal (red **x**) portions of the quality index are also offset for clarity. All vertical bars in the upper right panels represent one standard deviation, computed across all the BICAR runs. For the quality indices (right panels), mean and standard deviation for 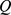 are shown (black symbols, upper right panels). 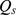 and 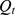 are shown without averaging; each BICAR run generates one pair of red and blue symbols (red and blue **x**, lower right panels).

Illustrated in Figure 5 are the reproducibility spectrum and reconstruction quality, averaged over ten independent BICAR runs, at each of seven different inverse signal-to-noise ratios for the BLOBS, SPEECH, and MUSIC data sets. Reproducibility values are always shown sorted in descending order. Since the problem setup is entirely symmetric (uniform addition of noise), absolute source order is meaningless; within each group no real source is easier or harder to extract than any other. Note that BICAR performs quite well even in the presence of moderate noise for all three data sets. The quality of reconstruction is quite poor at the highest noise levels (10 and 100-fold more noise than signal), but this is an extreme regime where good ICA performance will be hard to achieve. In all three data sets, BICAR is quite robust to small amounts of noise, and can even form reasonable reconstructions at a SNR of unity.

We have deliberately chosen challenging problems on which to test BICAR; BLOBS is relatively simple, but SPEECH and MUSIC are not. Variations in BICAR performance are not due only to added noise; for SPEECH and MUSIC (and the spatial part of BLOBS) each BICAR run used an entirely different set of sources. Different (random) images were created or selected and different (random) pieces of the audio recordings were extracted. No particular effort was made to ensure that the sources were always sparse, which is essentially the criterion FastICA is using to decompose the data. Indeed, the results for 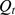 for SPEECH (panel B) and MUSIC (panel C) are somewhat surprising. Despite the fact that Blind Source Separation was originally developed for speech signals [13], and that the MUSIC sources, being noisy live recordings of rock bands, may in some sense already be mixtures, 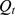 shows a very similar pattern in both SPEECH and MUSIC. In all, BICAR does remarkably well on quite challenging data sets.

In both SPEECH and MUSIC spatial source quality decays more slowly than temporal quality, while they are more symmetric in BLOBS. This is easy to understand given the amount of data available for estimating the temporal and spatial sources. BLOBS contains sixteen mixtures of five temporal sources and sixteen mixtures of five spatial sources, so the amount of data available for ICA to do the estimation in both the spatial and temporal case is identical. However, in SPEECH and MUSIC, there are still sixteen mixtures of five temporal sources, but 128 mixtures of five spatial sources. Hence there is much more redundant data available for spatial estimation, and the quality of extraction 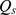 is subsequently higher over a wider range of added noise. SPEECH and MUSIC were not made as symmetric as BLOBS because real applications are quite unlikely to be nicely symmetric. For example, human neuroimaging typically involves roughly 100 temporal sensors but 800–1000 spatial time points, giving a ratio very similar to the 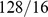 ratio in MUSIC and SPEECH.

Another feature of Figure 5 is the limited dynamic range in component reproducibility. In both BLOBS and SPEECH, all the sources are basically perfectly reproducible with little variance, except at the most extreme noise levels. Even in MUSIC, where there is more variation in component reproducibility, all five BICAR sources have 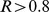, regardless of the value of 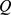 for the BICAR run. In the simplest data sets this high 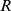 is easy to understand. FastICA produces different results from run to run because of local minima and convergence failures; it is inferences contaminated by these difficulties that we wish to guard against by using BICAR. In BLOBS, there may simply not be multiple minima; the signals are so simple that ICA reproduces the same decomposition every time, until the noise variance is much larger than the signal variance. However, the apparent decoupling of 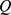 from 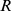 begs the question: is there a relationship between reproducible components and “correct” (true) ones?

**Figure 6:**
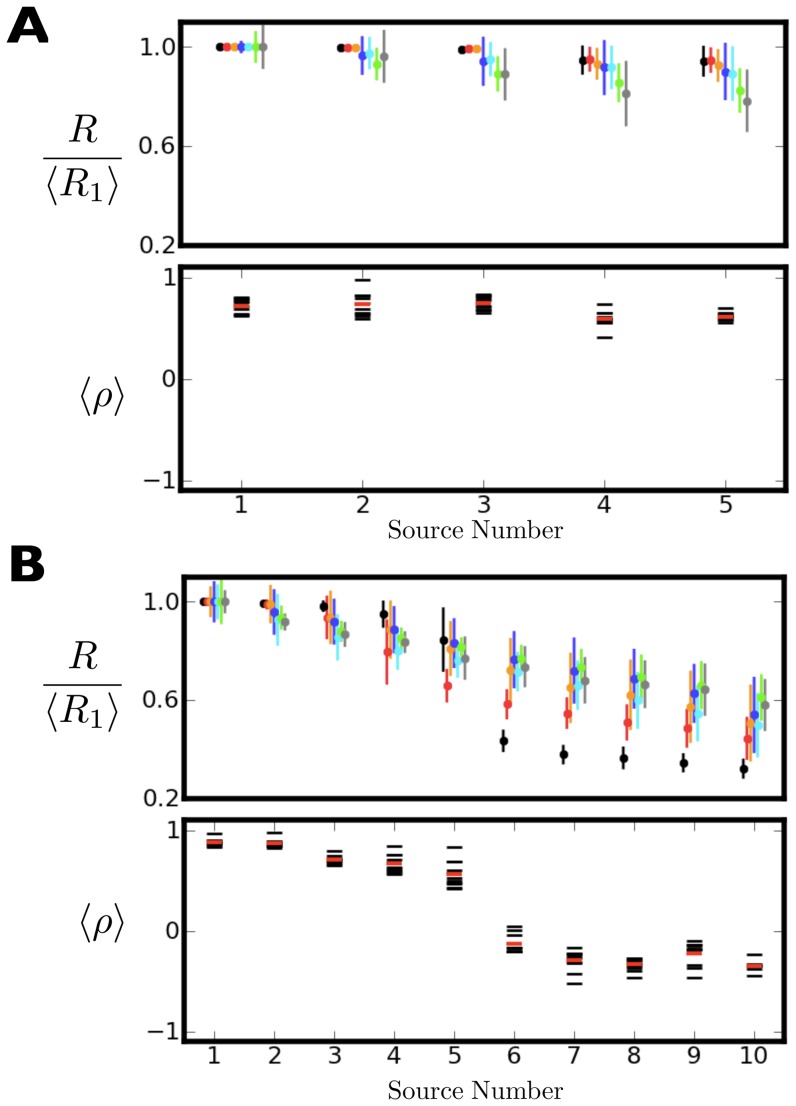
Mean reproducibility and correlations between component reproducibility and similarity to known sources using the MUSIC data. See text for definitions of these quantities. In the reproducibility plots (upper panels in **A**, **B**), sources have been rescaled so that the most reproducible source for each BICAR run at each noise level has reproducibility equal to unity. The lower panels in **A**, **B** show the correlation of reproducibility with known source similarity (see text), computed across all noise levels. The values for each of ten BICAR runs are black horizontal lines, and the mean over runs is shown in red. **A**. Five components were extracted in each BICAR run. The upper panel is identical to the left subpanel of figure Fig. 5C, with the exception of the rescaling already mentioned. Higher reproducibility is indeed correlated with similarity to a true source, but this is basically true for all five sources. **B**. Identical to **A**, except that ten sources were extracted in each BICAR run. There is greater dynamic range in the reproducibility spectrum than in **A**, particularly at low noise levels, where there is a clear break between sources five and six. As the noise increases, the reproducibility spectrum flattens out. The lower panel shows that less reproducible sources are less similar to true sources, with the second five sources (recall there are five true sources) having no consistent relationship to the known sources.

Figure 6 shows the answer to this question using the MUSIC data, and the answer is “yes.” As components become less reproducible, they look less and less like the true sources that comprise the mixture. Figure 6A (upper panel) repeats the left panel from Figure 5C, with all reproducibility spectra rescaled so that the most reproducible source in each set has 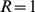. In Figure 6A (lower panel), the correlation between BICAR source reproducibility and similarity to a true source is shown. These similarity values are simply row maxima of the absolute correlation matrix between the BICAR sources and the true sources. In each case, these correlations are computed for a single BICAR run across all the added noise values. So, for instance, one set of BICAR simulations in Figure 6A at seven different noise values yields five correlations, one for each source. Each horizontal black bar denotes the result from one set of simulations, and the red bar marks the mean over ten sets. The sources have been sorted by decreasing reproducibility. One can see that there is indeed a high degree of correlation between reproducibility and true source similarity. However, this degree of correlation does not really fall off as the sources become less reproducible.

Figure 6B clarifies this result. In extracting five sources from our mixtures, we have incorporated all prior knowledge — we know the mixtures contain five true sources and we ask for five. However, in more realistic applications, the user will have no idea how many real sources there are and would have to use some method to estimate that number [34]–[36] or simply guess. Faced with this problem, one might simply ask for as many sources as possible (sixteen in this case), or at least close to that number. If the calculations in Figure 6A are repeated, but this time requesting ten sources (twice as many as truly exist) in each decomposition, Figure 6B is obtained. A dramatic change is immediately apparent. The dynamic range of the reproducibility spectrum increases, and for noiseless data (black circles) the 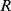 spectrum immediately tells us that there are only five sources actually present (Figure 6B, upper panel). As the noise increases, it becomes harder to immediately see the true number of sources present in the mixture.

There is a similarly dramatic change in the correlations between extracted and true components (Figure 6B, lower panel). The degree of correlation between reproducibility and source similarity falls off as components become less reproducible. In every case the five most reproducible BICAR components show strong correlations between 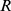 and similarity to the true sources, while the second five show no relationship. This demonstrates two principles. First, the more reproducible a BICAR component is, the more it resembles a true source, even as that overall level of similarity drops as the noise power increases. Second, even if the number of real sources cannot be clearly identified via a gap in the reproducibility spectrum, components should always be analyzed in order of decreasing reproducibility, as the larger the reproducibility the closer to a physical source that component will be. In certain real applications [26] there may be criteria that could be used to sort components; reproducibility should form an additional criterion.

**Figure 7:**
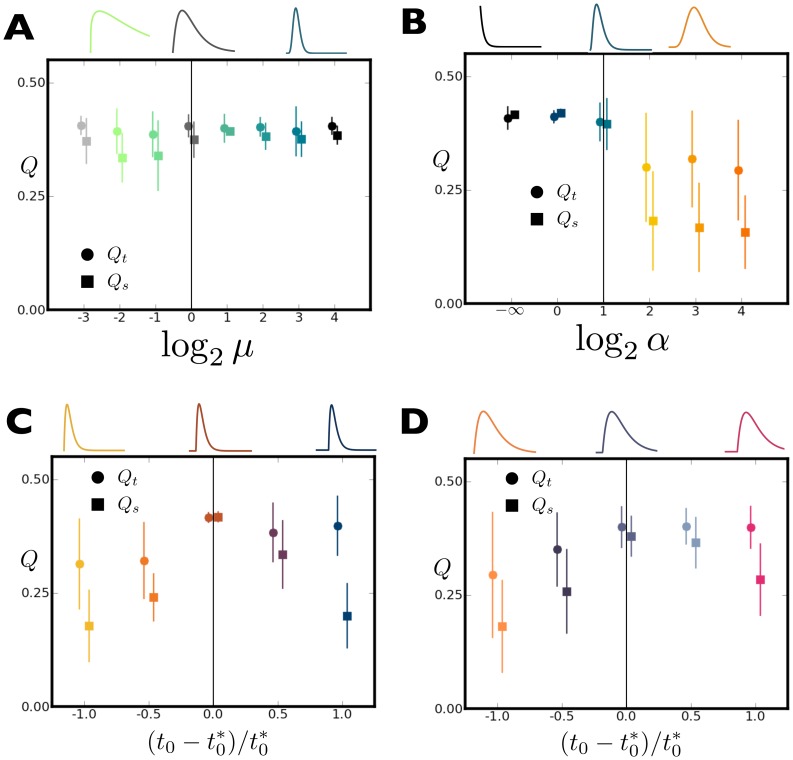
Effect of transfer function parameters on quality of BICAR reconstructions of the BLOBS data. Each of the four panels shows the mean and standard deviation of 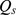 (filled circle) and 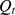 (filled square) over ten BICAR simulations. In all cases the mixing data was generated using one set of parameters for the transfer function, and BICAR was used to recover the true sources using another set of parameters. Each panel has a vertical line that indicates simulations with a perfect match between mixing and recovery parameters. In all cases the parametric transfer function given in Eqn. 9 was used. Sample transfer function shapes are shown above each panel, color coded to match the appropriate data point. **A**. Adjustment of function rise time and decay, keeping the location of the peak constant (see text). **B**. Adjustment of peak location with the decay rate (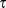) held fixed. Note the sharp decline in quality for a delayed peak. **C**. Adjustment of the lag parameter. Performance is plotted as a function of fractional difference in the 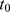 used for recovery versus the one used for generation. **D**. Here the lag parameter has been adjusted in an identical manner to **C**, but with wider transfer functions. Note that the loss of recovery quality seen in **C** is mitigated here by the wider transfer functions.

So far the transformation in Eqn. 5 has been assumed known. In most real applications the user has imperfect knowledge of the transfer function that connects the temporal sources with the spatial mixing coefficients. The parametric transfer function (Eqn. 9) employed in this study can be manipulated in several ways, and Figure 7 shows the results of mixing the data using one 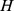 and running BICAR with a different transfer function.

- With 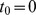, the function has a peak at 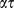. Both the rise time and decay rate of 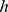 can be adjusted while keeping the location of the maximum constant. This is achieved via the transformation 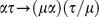, for variable 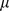 (Fig. 7A).
- With 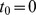, the location of the peak can be adjusted while keeping the asymptotic decay rate 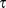 constant by simply adjusting 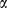 (Fig. 7B).
- The shape of 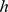 can be fixed and the delay 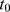 adjusted (Fig. 7C,D).

All BICAR runs in Figure 7 were performed on the BLOBS data at an SNR of unity. Reproducibility is not shown for any of these calculations; as demonstrated in Figure 5 the BLOBS data is simple enough that all extracted components are highly reproducible. This remains the case here (not shown). Figure 7A indicates robustness to the lack of knowledge about rise time and decay rate of the transfer function (note the logarithmic 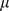 axis). However, if one is unsure about those parameters in a real situation, one is better off guessing the transfer function is sharper rather than broader. In Figure 7B, there is a much more pronounced asymmetry; there is little cost when reconstructing with a transfer function with a peak close to zero but a pronounced decay in quality for overshoots of the true the peak location. Figures 7C and D show the effects of changing 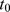 but keeping all other shape parameters constant. Here there is sensitivity to 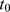 in both directions, although it should be noted that these ranges are rather extreme; they reflect up to 100% error in 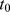 in both directions. Figure 7D illustrates that the filter width interacts with 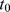; a larger range of 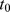 is tolerated when the transfer function is broader. In each case 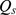 falls off more quickly than 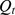, which is expected; the dominant effect of transfer function mismatch is pairing errors between the reconstructed sources.

As a practical guide to transfer function uncertainty, we would offer the advice to guess sharp and early; the results of Figure 7 show that BICAR is quite robust to certain types of misspecification in the transfer function. It is possible in principle to estimate the transfer function (either parametrically or as a set of filter coefficients) from within BICAR itself, using matching quality as an objective function. This area is outside the scope of this manuscript but is an extension of BICAR which we are actively studying.

## Discussion

We have presented a data-driven method that extracts reproducible pairs of spatial and temporal components from pairs of data sets with arbitrarily different spatial and temporal resolution. In cases where a credible model already exists for data assimilation [8]–[12], BICAR provides a complementary approach that is purely data-driven. In situations where a credible model is unknown, impossible, or suspect, an algorithm like BICAR may be the only option for joint mining and/or model reduction of such data.

BICAR is inspired by and shares methods with RAICAR [27] but improves and extends RAICAR in several ways. Most obvious is the pairing of spatial and temporal components in order to perform multiresolution data fusion. This opens up a set of powerful BICAR extensions already alluded to. One is the use of temporal source/spatial source matching quality as an objective function to optimize over a family of transfer functions, when the BICAR transfer function is unknown or poorly specified. Another is the choice between nondegenerate versus degenerate matching. While only nondegenerate matching was considered in this study, there may be advantages to allowing multiple temporal sources to match the same spatial source. This would be the case if ICA “oversplits” the temporal data such that one physical process is broken into several ICA components. These sources could be recombined if they match the same spatial source. It would also be useful to compare “all against all” matching, in which a temporal source from any of the 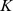 realizations could match a spatial source in any other realization, to the “online” matching considered in this manuscript, in which matching occurs between pairs of realizations, one pair at a time.

BICAR could also be expected to deal well with mismatches. This would occur when sources in one of the datasets have no true pair in the other dataset, as defined by Eqn. 5. With nondegenerate matching each source in the temporal data will be paired with a source in the spatial data, but mismatched sources would not be expected to pair reproducibly — repeatedly in many iterations. Therefore, these mismatched sources should end up near the low end of the reproducibility spectrum. This is as it should be, as BICAR is designed to find *paired* reproducible sources, not simply two sets of reproducible sources with no relation to one another.

In the process of developing BICAR, important modifications to the published RAICAR algorithm have also been made. For one, the definition of reproducibility, and the way in which sources are averaged to obtain BICAR components, differ from RAICAR [27]. The way these issues are handled in BICAR ensures that *all* sources from *all* ICA realizations are used to calculate reproducibility and construct the BICAR components. This is obtained via favoring the weighted averaging scheme described in Algorithm, as opposed to selective averaging of only components passing some similarity threshold. In addition, the sign canonicalization step, while simple, is absolutely necessary to enable the use of BICAR on a broad range of data. Sign reversals of the type described in Algorithm are ubiquitous and, if ignored, lead to nonsensical BICAR components. This seems to be particularly true when performing ICA on temporal data.

Extremely stringent tests have been set for BICAR in this manuscript. While BLOBS, SPEECH, and MUSIC are of relatively low dimension (five sources), much of the data that produces them comes from real world signals — audiobooks, astronomical images, and recorded music. No particular randomly drawn data set is guaranteed to be easily ICA-decomposable even in the low noise case, so our performance measures study ensembles both of different signals and increasing noise levels. Finally, the way exogenous noise has been added makes for a difficult problem as the noise level increases. Our measures of good performance demand that *all* BICAR sources be close to the true sources. Since ICA decompositions reproduce the data matrix with minimal error, the added noise must go somewhere, either into the mixing elements or the sources. Hence it would be very difficult for us to obtain high 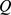 values at all noise levels. If instead some sources were made noisier than others — effectively adding the noise directly to the sources and not the mixtures — the least noisy sources could have potentially been extracted at much higher noise levels.

For neuroimaging applications, no epoching, trial averaging, or statistical parametric mapping [40] is required to run BICAR. This means that decomposition and reproducibility calculation are completely decoupled from experimental design. One can therefore employ this task information at the end of the process to construct component ranking criteria that are independent of, and can be used in tandem with, reproducibility.

The transfer function that links the two data sets is particularly relevant for human neuroimaging [39], an application area in which we are interested [7]. However, the function is quite simple and generic, and essentially stands in for any delayed low-pass filter. Using a parametric transfer function has made it easy to study how robust BICAR is to transfer function misspecification. Even without attempting to estimate the transfer function from within BICAR, BICAR has good robustness properties to relatively large transfer function errors in both location and shape parameters.
